# Hepatic steatosis associated with decreased β-oxidation and mitochondrial function contributes to cell damage in obese mice after thermal injury

**DOI:** 10.1038/s41419-018-0531-z

**Published:** 2018-05-10

**Authors:** Li Diao, Christopher Auger, Hisato Konoeda, Ali-Reza Sadri, Saeid Amini-Nik, Marc G. Jeschke

**Affiliations:** 1 Sunnybrook Research Institute, Toronto, ON Canada; 2 Department of Surgery, Division of Plastic Surgery, Division of General Surgery, Department of Immunology, University of Toronto, Toronto, ON Canada; 3 Ross Tilley Burn Centre, Sunnybrook Health Sciences Centre, Toronto, ON Canada

## Abstract

Severely burned patients who are morbidly obese have poor clinical outcomes with aggravated metabolic consequences, a higher incidence of multiple organ dysfunction/failure, and significantly increased morbidity and mortality. The underlying mechanisms of these adverse outcomes are essentially unknown. Since the liver is one of the central metabolic organs, we hypothesized that thermal injury in obese patients leads to substantially increased lipolysis, hepatic fat infiltration, resulting in profound hepatic cellular and organellar alterations, consequently causing liver damage and severely augmented metabolic dysfunction. We tested this hypothesis using an obese mouse model subjected to a 20% total body surface area burn injury. C57BL/6 mice were randomly divided into low-fat diet (LFD) and high-fat diet (HFD) sham and burn groups (*n* = 6 per group) and fed for 16 weeks. 7 days after the thermal injury portal and cardiac blood were taken separately and liver tissue was collected for western blotting and immunohistochemical analysis. Gross examination of the liver showed apparent lipid infiltration in HFD fed and burned mice. We confirmed that augmented ER stress and inhibition of Akt-mTOR signaling dysregulated calcium homeostasis, contributed to the decrease of ER–mitochondria contact, and reduced mitochondrial β-oxidation in HFD fed and burned mice, leading to profound hepatic fat infiltration and substantial liver damage, hence increased morbidity and mortality. We conclude that obesity contributes to hepatic fat infiltration by suppressing β-oxidation, inducing cell damage and subsequent organ dysfunction after injury.

## Introduction

Modern civilization features redundant access to food supply and thus excessive caloric intake, which is the leading cause of pandemic obesity^1^. Consequently, clinicians are seeing more and more obese patients than ever in history. Even though there is an increased incidence of certain comorbid health problems (such as diabetes, hypertension, sleeping dyspnea) in obese people, their health is generally unaffected unless challenged by additional insults such as trauma, infection, etc. Indeed, multiple clinical studies have shown higher morbidity and mortality rates in obese patients upon acute injury or sickness, although the underlying mechanisms are largely unclear^2–4^. Compared to the wealth of knowledge of metabolic derangements in either obesity (i.e., diabetes)^5^ or post-trauma^6^, there is a dearth of literature concerning the pathology of severe trauma in the obese population. More interestingly, our previous clinical observations showed that mild obesity is beneficial, whereas morbid obesity is detrimental to trauma victims^7^. Hence, there is a fascinating conflict in terms of what is different about mild vs. morbid obesity. We therefore asked what the underlying mechanisms by which severe obesity worsens clinical outcomes are.

Since the liver is the central metabolic organ, we hypothesized that the augmented effect of chronic hepatic stress as a result of obesity^8–10^ in tandem with the acute perturbation of homeostasis post-trauma^11,12^ would contribute to worse clinical outcomes in this group of patients. We sought to test this hypothesis using a mouse model of high-fat diet (HFD)-induced morbid obesity plus a major burn covering 20% of the total body surface area (TBSA).

## Results

### HFD and burn leads to hepatic fat infiltration and increased lipolysis

**Fig. 1:**
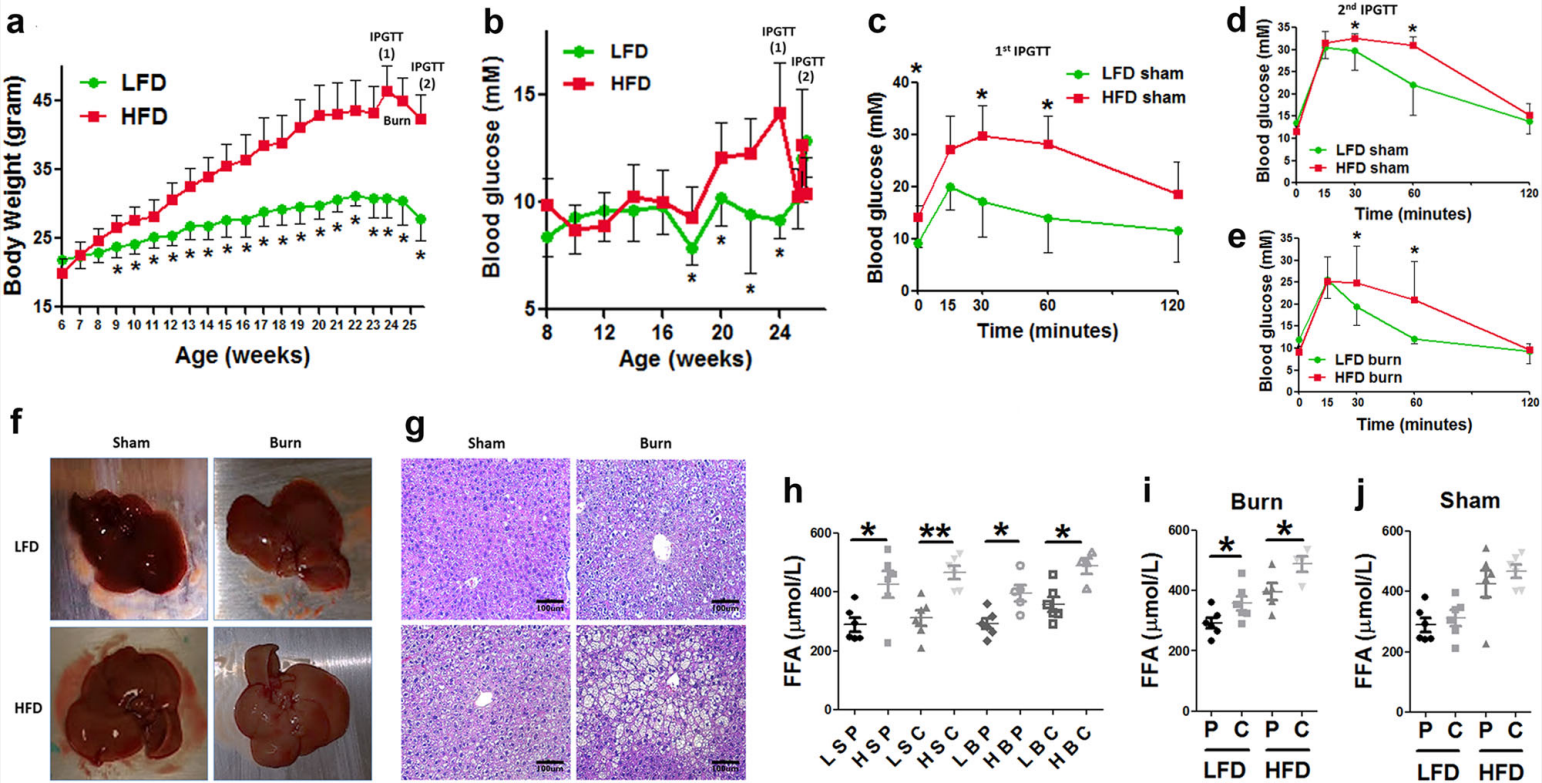
Augmented hepatic fat infiltration in obese mice after thermal injury. Weight gains (**a**), level of blood glucose (**b**), and IPGTT before the burn injury (**c**) was compared between mice fed with LFD or HFD. IPGTT was also performed in LFD and HFD mice 6 days after sham burn (**d**) and burn injury (**e**). Representative gross pathology images of the whole livers taken from different treatment groups (**f**) were presented alongside representative images of H&E staining of mice liver tissue from different groups (**g**). Plasma level of FFA in blood taken from portal vein and central vein in different treatment groups were compared among LFD vs. HFD animals (**h**). Plasma levels of FFA in blood taken from portal vein vs. central vein were also compared in shams (**i**) and burned mice (**j**). The data are presented as means ± SEM. Statistical analysis was performed using a two-tailed Student’s *t* test with *P < 0.05 considered statistically significant from (**a**–**e**) and one-way ANOVA followed by Bonferroni’s post hoc test was performed for (**h**–**j**) with *P < 0.05 considered statistically significant and **P < 0.01. *N* = 12 animals per group for (**a**–**c**), *N* = 6 animals per group for (**d**–**j**)

On the basis of well-accepted murine model of HFD-induced obesity^13^, we fed the mice with either HFD or low-fat diet (LFD) for 16 weeks and we observed significantly higher body weight gain in the HFD group vs. the LFD group by the end of the 16 weeks of feeding (Fig. 1a). Concomitant elevation of blood glucose level was observed in HFD mice (Fig. 1b, *p* < 0.05). We also conducted an intraperitoneal glucose tolerance test (IPGTT) and found the impaired glucose clearance in HFD mice (Fig. 1c) confirming metabolic alterations.

We then applied a thermal injury of 20%TBSA^14^ by the end of the 17th week after the initiation of the feeding. We ended the study 6 days after the thermal injury and conducted another IPGTT, which showed impaired blood glucose clearance at 30 min and 1 h after the intraperitoneal injection of dextran (Fig. 1d, e). These IPGTT results indicate increased insulin resistance in the obese mice both in sham and post-burn.

Gross examination of the liver at the end of the study revealed some yellow/pink discoloration of the liver in HFD sham and LFD burn animals. This color change was even more pronounced in the HFD burned mice (Fig. 1f), indicating increased hepatic fat infiltration; this finding was confirmed by H&E staining, which clearly shows increased fat infiltration of the liver in HFD or burned animals, with the greatest fat infiltration in HFD burned animals (Fig. 1g).

We hypothesized that increased hepatic fat infiltration is due to increased levels of circulating free fatty acids (FFA). When comparing plasma levels of FFA, we observed elevated FFA in HFD vs. LFD mice regardless of the comparison between portal and central vein blood samples or between shams and scald burned animals (Fig. 1h, *p* < 0.05). More importantly, we observed significantly higher level of FFA in central vein blood than that in portal vein blood in burned mice (Fig. 1i, *p* < 0.05) but not in shams (Fig. 1j). Such significantly elevated FFA in central vein blood indicates increased lipolysis of the peripheral adipose tissue after thermal injury^15^. These observations indicated that a HFD followed by a burn injury is associated with increased lipolysis and circulating FFA, resulting in an increased pre-load of lipids to the liver contributing to substantial hepatic fat infiltration.

### De novo lipogenesis is not activated in HFD mice after thermal injury

**Fig. 2:**
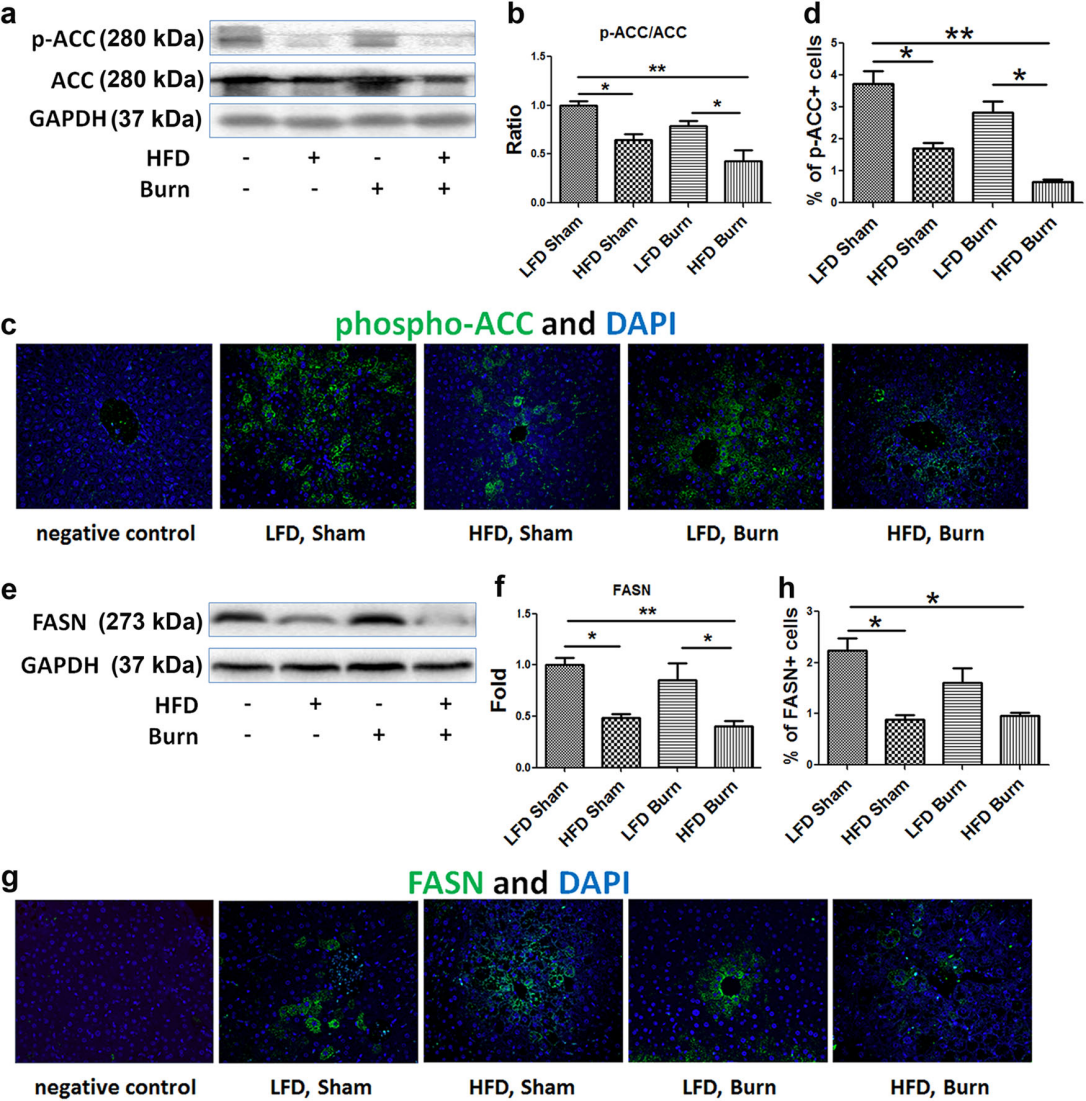
Repression of de novo lipogenesis in HFD mice after thermal injury. Representative images (**a**) and quantitative densitometric analyses (**b**) of the Western blot of phospho-ACC (Ser79) and ACC were presented alongside immunofluorescent staining of phospho-ACC (**c**, magnification ×200) and percentage of phospho-ACC positive cells (**d**) in liver tissue. Representative images (**e**) and quantitative densitometric analyses (**f**) of the western blot of FASN were presented alongside immunofluorescent staining of FASN (**g**, magnification ×200) and percentage of FASN positive cells (**h**) in liver tissue. Data are presented as means ± SEM. *P < 0.05 and **P < 0.01. *N* = 6 animals per group

To examine how increased FFA pre-load was associated with fat infiltration of the liver, we hypothesized and determined if there was an increase in de novo lipogenesis commonly considered as the significant source of intra-hepatocellular lipids in fatty liver diseases^16^. Western blot analysis showed the level of liver tissue inhibitory phospho-acetyl CoA carboxylase (Ser79) (p-ACC) was significantly decreased in HFD-fed mice with or without burn injury as compared with LFD sham (Fig. 2a, b, *p* < 0.05), implicating increased conversion from acetyl-CoA to malonyl-CoA in HFD-fed mice. However, the level of hepatic fatty acid synthase (FASN), the key rate-limiting enzyme of de novo lipogenesis^17^, was significantly decreased in HFD fed mice, especially in HFD burned group (Fig. 2e, f, *p* < 0.01), indicating that there is no activation of lipogenesis albeit the possible increased substrate pressure of malonyl-CoA. There were no significant changes in liver tissue level of p-ACC and FASN in LFD burned mice as compared with LFD shams, indicating that burn alone has no significant impact on hepatic lipogenesis. To further confirm this finding, we performed immunofluorescent staining of p-ACC and FASN in the liver tissue sections (Fig. 2c, g). Statistical analysis of the positive cell counts demonstrated results consistent with the Western blot analyses (Fig. 2d, h). Hence, despite the increased FFA pre-load, there is no evidence supporting the significant increase in the de novo lipogenesis in the liver tissue after HFD and/or thermal injury.

### Decreased hepatic lipid β-oxidation and attenuated mitochondrial electron transport chain (ETC) function associated with hepatic fat infiltration

To investigate the mechanisms of the increased hepatic fat infiltration, we asked next whether it can be attributed to reduced lipid oxidation. We measured the expression of hepatic carnitine palmitoyltransferase-1 (CPT1A), which is the rate-limiting enzyme for liver mitochondrial β-oxidation, translocating fatty acids across the mitochondrial membrane. The level of the expression of CPT1A would thus reflect the hepatic β-oxidation activity^18^.

**Fig. 3:**
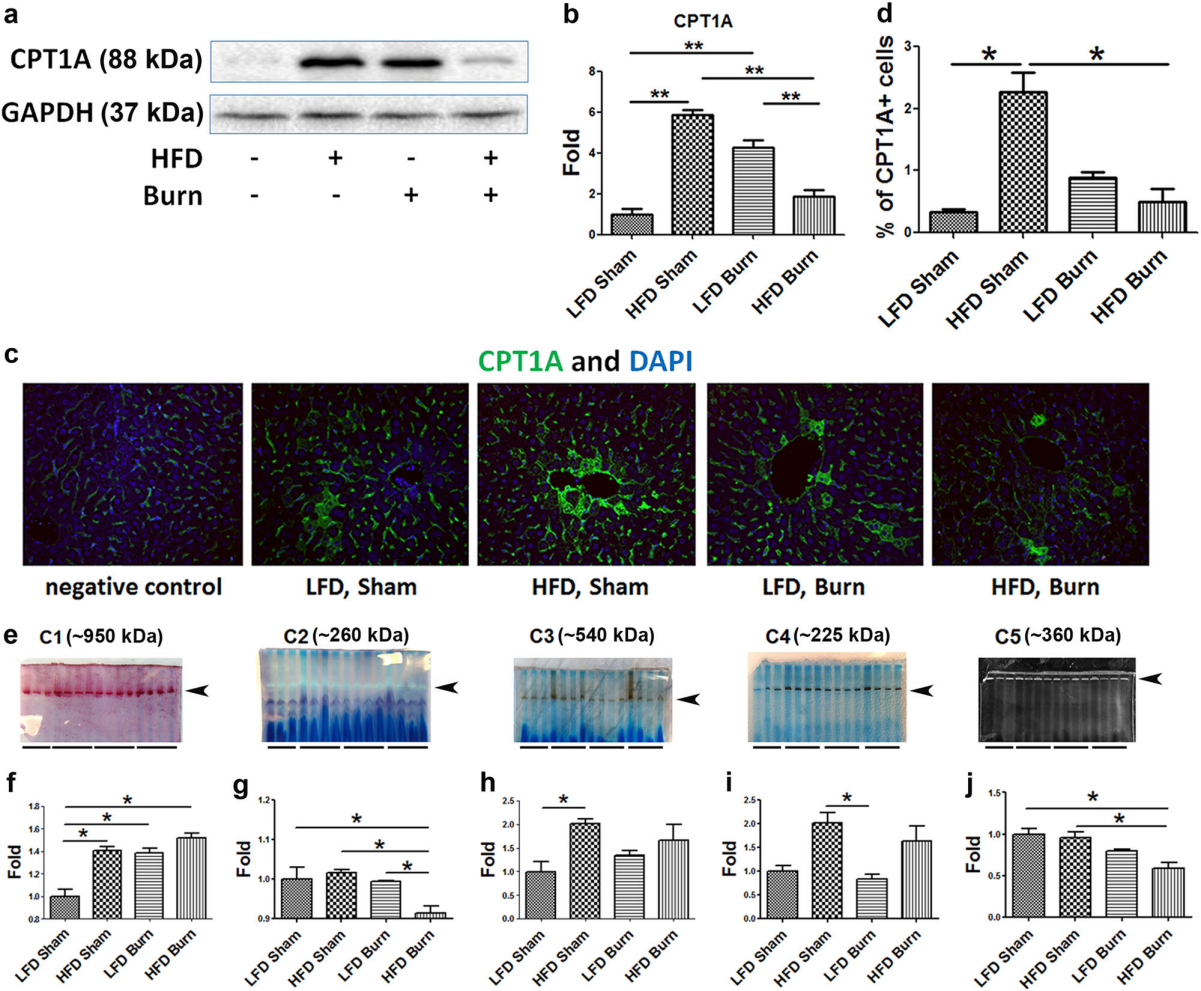
Impaired hepatic lipid oxidation and mitochondrial electron transport chain (ETC) activities in the obese mice after thermal injury. Representative images (**a**) and quantitative densitometric analyses (**b**) of the western blot of CPT1A were presented alongside immunofluorescent staining of CPT1A (**c**, magnification ×200) and percentage of CPT1A positive cells (**d**) in liver tissue. Representative images (**e**) of native polyacrylamide gel electrophoresis followed by in-gel activity assays for mitochondrial ETC complexes I, II, III, IV and V in the liver tissue were presented together with the quantitative densitometric analyses for the in-gel blots (**f**–**j**). The data are presented as means ± SEM. *P < 0.05 and **P < 0.01. *N* = 6 animals per group

Western blot analysis showed increased expression of CPT1A in HFD shams and LFD burned mice as compared with LFD sham (Fig. 3a, b, *p* < 0.01), but not in HFD burn animals. Such an increase in CPT1A in HFD sham and LFD burn is consistent with other studies showing that hepatic mitochondrial β-oxidation is enhanced in the liver of genetically obese (ob/ob) mice^19^, HFD-fed rats^20^ as well as in patients with steatohepatitis^21^, implicating the increased substrate pressure and activation of the compensatory mechanisms of lipid turnover such as hepatic peroxisome proliferator-activated receptor alpha (PPARα). However, there was a significantly lower level of CPT1A in HFD burned animals as compared with that of HFD only or burn only group. It is interesting to note the divergent response of lipid metabolism between HFD sham and HFD burned animals. While there is likely an increase in malonyl-CoA in both groups due to the activation of ACC, it seems that in HFD sham mice, significantly activated lipid β-oxidation might be compensatory enough to limit the magnitude of the fat infiltration, whereas in HFD burned mice, the accumulation of the malonyl-CoA might be overwhelming and significantly inhibit β-oxidation, contributing to augmented hepatic fat infiltration^22^.

To confirm our western blotting data, we performed immunofluorescent staining of CPT1A in liver tissue sections. We noticed stronger positive signals of CPT1A in HFD shams and LFD burned mice as compared with LFD shams (Fig. 3c) and statistical analysis of the positive cell counts demonstrated results that are consistent with western blot analyses (Fig. 3d). This suggests that in HFD burned animals, the liver is not able to β-oxidize the significantly increased inflow of FFA from peripheral lipolysis, resulting in hepatic accumulation of FFA.

To further investigate the underlying mechanisms of impaired hepatic lipid metabolism in HFD burned mice, we analyzed the mitochondrial ETC activities in line with the changes in the hepatic mitochondrial lipid β-oxidation^23^. The increase of complex I and complex III activity in HFD groups implies that there may be an increase in ROS production as these are the primary sites of superoxide formation. Moreover, the lowered activity of ATP synthase in the HFD burned group is particularly detrimental, as it suggests impaired energy formation in this cohort of mice (Fig. 3e–j)^24,25^. Furthermore, a significant decrease of the Complex II activity not only reinforced the likelihood of impaired mitochondrial energy production and increased ROS production, but also implicated cell damage in HFD burned animals^26^.

### Perturbed inter-organelle Ca^2+^ homeostasis correlated with decreased ER–mitochondrial contact

**Fig. 4:**
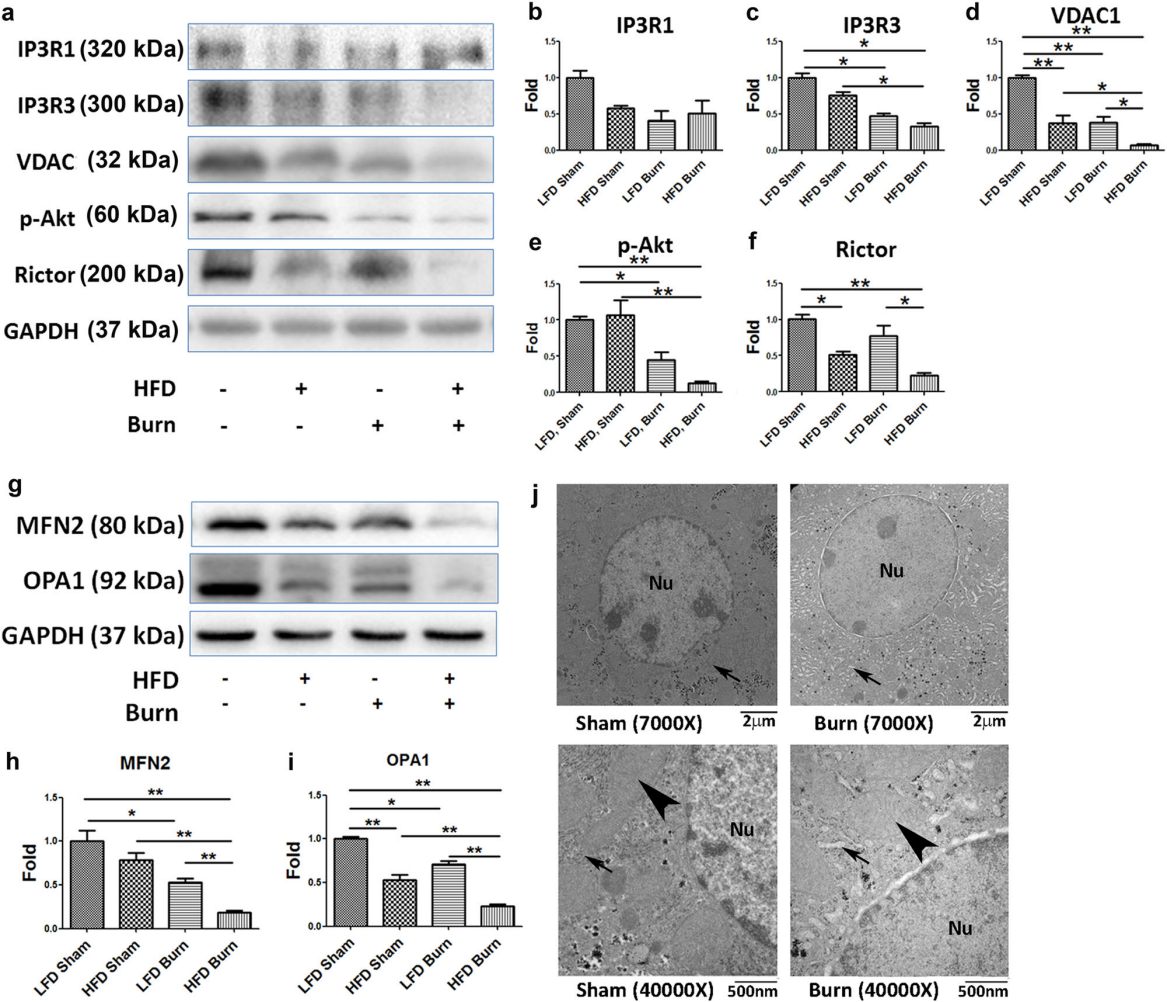
Mitochondrial metabolic dysfunction is correlated with the decrease of ER–mitochondrial contact, implicating the perturbed inter-organelle Ca^2+^ homeostasis in the liver of obese mice after thermal injury. Representative images (**a**) were presented with the quantitative densitometric analyses (**b**–**f**) of Western blots for IP3R1, 3, VDAC1, phospho-Akt (Ser473), and Rictor in the liver tissue. Representative images (**g**) and quantitative densitometric analyses (**h**, **i**) of western blots for MFN2 and OPA1 implicate the changes in mitochondrial dynamics and structure, which was demonstrated by TEM (**j**). Nu marks nucleolus. Arrows indicate the space between mitochondria and ER; arrowheads point to mitochondrial cristae structure. Data are presented as means ± SEM. *P < 0.05 and **P < 0.01. *N* = 6 animals per group

Considering the importance of Ca^2+^ homeostasis in mitochondrial bioenergetics, we sought to investigate whether there were perturbation in ER–mitochondrial Ca^2+^ homeostasis in HFD and/or burned mice as compared with LFD sham by checking several key regulators of mitochondrial Ca^2+^ channels, including inositol 1,4,5-triphosphate receptor 1 (IP3R1), IP3R3, Voltage-dependent anion channel 1 (VDAC1), p-Akt (Fig. 4a)^27,28^. While there were no significant changes in IP3R1 among the groups (Fig. 4b, *p* > 0.05), there was a significant decrease of IP3R3 levels in burned mice as compared with LFD sham (Fig. 4c, *p* < 0.05). Since IP3R3 is the major channel for Ca^2+^ efflux from ER to mitochondria under mild stress conditions^29^, decreased IP3R3 implicated lower Ca^2+^ levels in mitochondria due to insufficient Ca^2+^ influx from the ER. Also, considering that VDAC1 is a multi-functional channel involved in Ca^2+^ and metabolite transport, energy production and in ER–mitochondria structural and functional association^30^, significantly decreased VDAC1 in HFD sham and LFD burned mice not only indicated the possibility of decrease in mitochondrial mass but also implicated the derangement of Ca^2+^ transport between ER and mitochondria (Fig. 4d, *p* < 0.01). Nevertheless, in HFD burned mice, significantly decreased phospho-Akt (Ser473) and Rictor (Fig. 4e, f, *p* < 0.05) indicated impairment of Akt-mTORC2 signaling, while it has been well accepted that inhibition of Akt-mTORC2 signaling and subsequent phosphorylation of IP3R in general account for the depletion of Ca^2+^ from ER to mitochondria, thus triggering cell death pathways^31^.

More importantly, with the growing appreciation of the ER–mitochondrial axis, we postulated that a decrease of ER–mitochondria contact in HFD and/or burned mice would contribute to the aforementioned perturbed inter-organelle Ca^2+^ homeostasis and mitochondrial energy production. Since mitofusin 2 (MFN2) is widely accepted as the major regulator of the mitochondria–ER contact^32,33^, and its coupling molecule, OPA1 is the key regulator of mitochondrial inner membrane fusion and cristae structuring^34^, we performed western blot analysis on the two proteins (Fig. 4g). A significant decrease of MFN2 in burned mice indicated decreased ER–mitochondrial contact after burn injury. A further decrease of ER–mitochondrial contact was implicated in HFD burned mice as compared with either HFD sham or LFD burn (Fig. 4h, *p* < 0.01). A similar pattern was noted for OPA1 levels (Fig. 4i, *p* < 0.01). These findings in western blot analysis were confirmed via transmission electron microscopy (TEM) (Fig. 4j). As compared with sham, increased space among the ER and mitochondria (arrows) as well as the shrinkage of the mitochondrial cristae (arrowheads) were evident in the liver tissue sections of the burned mice.

### Augmented hepatic ER stress, inflammasome activation and aggravated cell damage in HFD mice after thermal injury

**Fig. 5:**
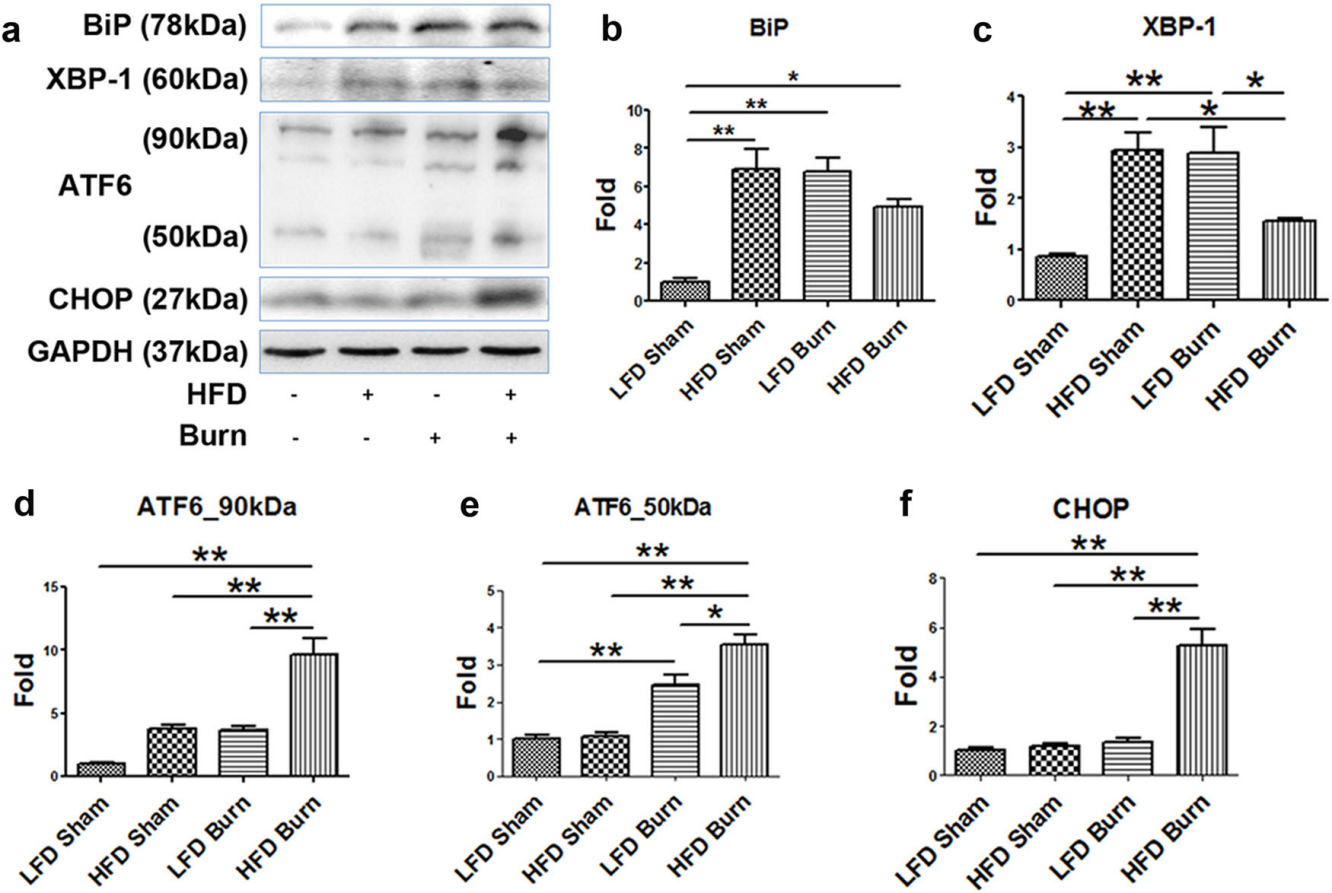
Augmented hepatic ER stress in HFD burned mice. Representative images (**a**) were presented with the quantitative densitometric analyses (**b**–**f**) of Western blot for ER stress markers of BiP, XBP-1, ATF6, and CHOP in liver tissue. Data are presented as means ± SEM. *P < 0.05 and **P < 0.01. *N* = 6 animals per group

**Fig. 6:**
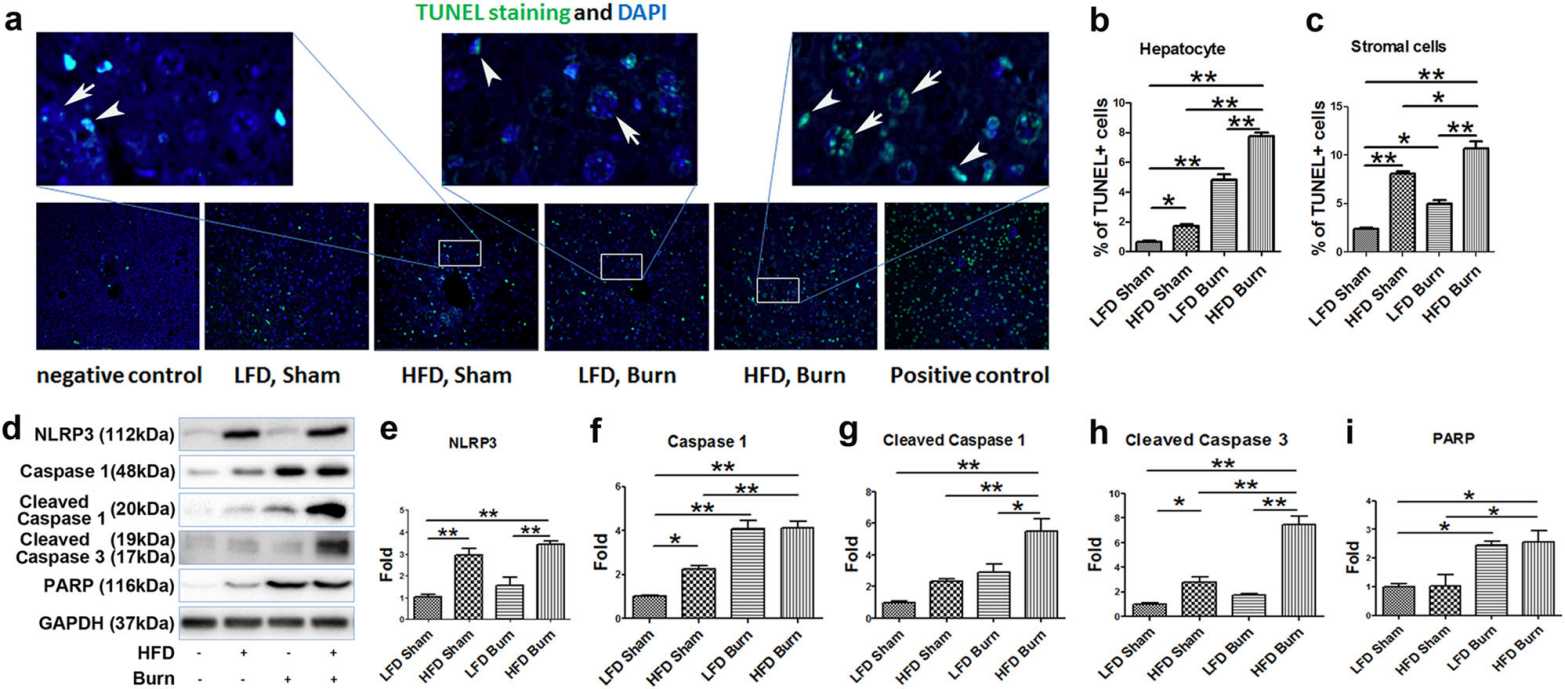
Increased liver cell apoptosis correlates with NLRP3 inflammasome activation, enhanced pro-apoptotic signaling and DNA damage in HFD burned mice. TUNEL and DAPI staining of the liver tissue (**a**) were presented together with the percentage of TUNEL positive hepatocytes and stromal cells (**b**, **c**). Arrows indicate apoptotic hepatocytes and arrowheads indicate apoptotic stromal cells (magnification ×400 in the upper panel, ×200 in the lower panel). Representative images (**d**) were presented with the quantitative densitometric analyses of western blot for NLRP3, caspase 1, cleaved caspase 1, cleaved caspase 3 and PARP in the liver tissue (**e**–**i**). The data are presented as means ± SEM. *P < 0.05 and **P < 0.01. *N* = 6 animals per group

With the above evidence of the changes in ER–mitochondrial structure, function and contact, it was reasonable to further postulate a concomitant cellular stress response in HFD and/or burned animals^35^. We thus determined the level of several important hepatic ER stress and subsequent unfolded protein responses (UPR) markers^36^. western blots (Fig. 5a) and densitometric analysis demonstrated augmented ER stress in the liver of HFD and/or burned animals. We observed the following two patterns of UPR upon the HFD and/or burn injury: (1) the significant activation of hepatic BiP and XBP-1 in either HFD sham or LFD burn group (*p* < 0.01), whereas less significant (BiP) or no significant change (XBP-1) in HFD plus burn group as compared with LFD sham control (Fig. 5b, c); (2) the level of ATF6 and CHOP increased significantly in HFD plus burn group (Fig. 5d–f, *p* < 0.01). Considering that CHOP is a pro-apoptotic transcription factor, we then sought for the evidence of cell damage upon HFD and/or thermal injury^37^. TUNEL staining of liver tissue sections confirmed the aggravated apoptosis in HFD sham, LFD burn and HFD burn groups as compared with LFD sham (Fig. 6a). Interestingly, we noticed that hepatocyte apoptosis was more severe in burned mice (Fig. 6b, sham vs. burn, *p* < 0.01), whereas stromal cell apoptosis was more significant in HFD treatment (Fig. 6c, LFD vs. HFD, *p* < 0.01). Nevertheless, a significantly higher rate of apoptosis was seen in HFD burned mice when compared to LFD shams and either intervention of HFD or burn alone (*p* < 0.01).

We postulated the increased inflammasome activation that contributes to the apoptosis of the stromal cells^38^. This was confirmed by the western blot of NOD-like receptor, pyrin domain containing 3 (NLRP3) and Caspase 1 (both total and cleaved form) in the liver tissue (Fig. 6d–g, *p* < 0.01). Also, elevated levels of cleaved Caspase 3 and Poly ADP ribose polymerase (PARP) were also consistent with the TUNEL staining, demonstrating the aggravated cell damage particularly in HFD burned mice (Fig. 6h, *p* < 0.01; 6i, *p* < 0.05).

## Discussion

In the current study, we aimed to determine why patients with obesity have altered metabolism and decreased survival after burn. We focused on liver pathology and used a mouse model of HFD-induced morbid obesity and 20% TBSA scald burn. The thermal injury model is well described, but we ensured adequate hypermetabolic and inflammatory responses when combining it with a high-fat diet-induced obesity.

In this model, we found profound hepatic fat infiltration in HFD burned mice, which is mainly attributable to increased lipolysis and impaired hepatic lipid β-oxidation and correlates with liver damage. It is very interesting to note that increased hepatic infiltration is not due to increase de novo lipogenesis. To investigate the underlying mechanisms of such metabolic impairment and tissue damage, we compared the hepatic ER stress responses, changes in mitochondrial ETC activities, ER–mitochondria communication, inflammasome activation and apoptosis signaling among sham, burn and HFD plus burn groups. We noticed that with a burn injury alone the hepatic responses are generally including the activation of ER UPR of increased expression of BiP and XPB-1^39,40^, and higher levels of CPT1A for increased β-oxidation. Mitochondrial ETC activities and ATP synthesis are also well maintained and inflammasome activation and apoptosis are mildly increased. Nevertheless, at least 2 phenomena implicated metabolic derangement after burn injury as compared with sham animals: 1) decreased mitochondria mass and perturbation of mitochondrial Ca^2+^ homeostasis as is indicated by the changes in IP3R3 and VDAC1; 2) decreased ER–mitochondria contact and mitochondrial dynamics as is manifested by the changes in the levels of MFN2 and OPA1 and morphological alterations seen in TEM. With the onset of ER stress, to chaperone the increased nascent, unfolded or misfolded proteins, BiP tends to dissociate from IP3R1 which is the major channel of Ca^2+^ flux from ER to mitochondria under physiological conditions, leading to inhibition of the IP3R1 Ca^2+^ channel. Moreover, a significant decrease of the IP3R3 level as seen in burned animals may also have an impact by decreasing mitochondrial Ca^2+^ levels. This is consistent with the decreased ATP synthase activity, decreased ER–mitochondria contact, and therefore, decreased energy production and, possibly concomitant increase in ROS production^41^. Furthermore, derangement of mitochondrial dynamics as was manifested by the changes in MFN2 and OPA1 links impaired energy production with cell damage^34^.

In HFD-induced obese mice after burn injury, drastic changes in almost every aforementioned cellular process brought about a significantly different outcome as compared with burn injury alone. Augmented ER stress was beyond the cellular capacity of molecular chaperoning and pro-apoptotic signaling was activated as was indicated by significantly increased levels of CHOP. In contrast to the inhibition of Ca^2+^ efflux from hepatic ER to mitochondria in LFD burned mice, there might be dysfunctional Ca^2+^ transport from ER to mitochondria due to significant activation of IP3R which is termed IP3-induced Ca^2+^ release^42^. We hypothesize that increased mitochondrial Ca^2+^ levels contribute to further impairment of energy production and aggravated cell damage^43^. Importantly, a greater decrease of ER–mitochondrial communication in HFD burned mice was seen with the concomitant and significant suppression of OPA1 and inhibition of mitochondrial ETC complexes activities, indicating impairment of mitochondrial energy production, and ultimately, aggravated cell damage^44^.

**Fig. 7:**
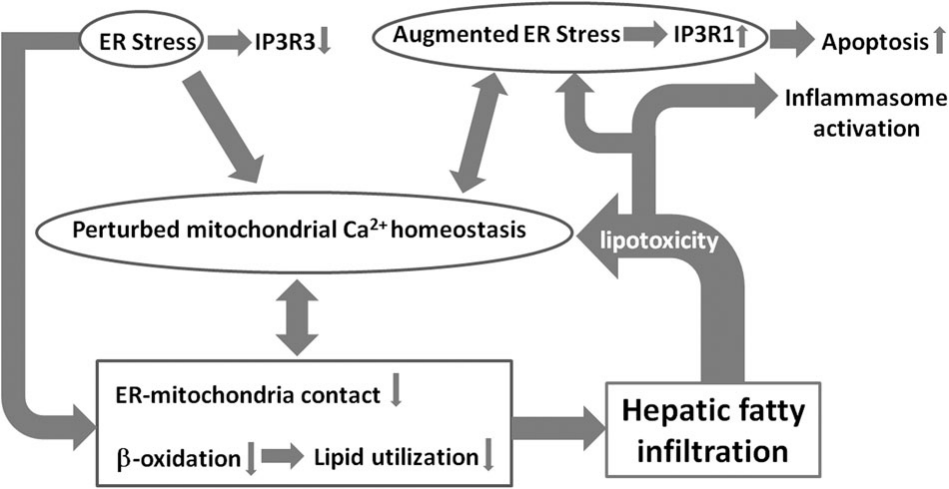
Hepatic fat infiltration is attributable to the vicious cycle of ER stress, mitochondrial dysregulation and cell damage in HFD burned mice. Hepatic ER stress and disturbed ER–mitochondria communication lead to the derangement of mitochondrial ETC activities, energy production, and impaired lipid metabolism. This contributes to the increased hepatic fat infiltration, which, together with hepatic inflammasome activation, results in increased liver damage in HFD burned mice

In summary, in LFD mice, burn injury stimulates an ER stress response with the increased likelihood of a lower level of mitochondrial Ca^2+^ and subsequent decreased ATP and increased ROS production, as well as decreased ER–mitochondrial contact, all correlated with a mild lipid infiltration. In HFD shams, there is mitochondrial fragmentation and lipid accumulation in the liver albeit compensatory increase of lipid β-oxidation. This is accompanied with NLRP3 inflammasome activation and increased apoptosis as compared with LFD shams. When HFD mice were challenged by burn injury, augmented ER stress-induced pro-apoptotic signaling; mitochondrial energy production was significantly impaired with further perturbed Ca^2+^ homeostasis; uncompensated impairment of ER–mitochondrial contact, faulty β-oxidation, and inflammasome activation occurred, leading to significantly decreased lipid turnover. Subsequent fat infiltration would aggravate ER stress, forming a vicious cycle and leading to liver organ damage (Fig. 7). As such, in animals with obesity and severe burn, it is the impairment of multiple cellular processes which compound upon each other that deregulates lipid homeostasis and, as a consequence, worsens outcomes for obese trauma victims. Accordingly, early and effective interventions to attenuate ER stress, inflammasome activation, and ROS production, as well as treatment to stimulate mitochondrial dynamics and restore mitochondrial Ca^2+^ homeostasis would be beneficial to this group of patients.

## Materials and Methods

### Animal model

Animal experiments were approved by the Animal Care and Use Committee of Sunnybrook Research Institute (AUP #467) in Toronto, ON. The National Institutes of Health Guidelines for the Care and Use of Experimental Animals were met. 6-week-old male C57BL/6 mice were purchased from The Jackson Laboratory (ME, USA) and were randomly chosen to receive HFD (TD.06414, Harlan Laboratories, WI, USA) to induce obesity; mice were fed low-fat diet (LFD, TD.08806, Harlan Laboratories, WI, USA) as control. After 16 weeks of feeding, IPGTT was performed by intraperitoneal injection of 20% glucose solution (2 g glucose per kg body weight) after overnight fasting followed by blood glucose measurement (Accu-Chek test strips, Roche, USA) at 0, 15, 30, 60 and 120 min after the glucose intraperitoneal injection. The animals in each group were sub-divided into sham and burned groups (*N* = 6 in each group). HFD/LFD and water was given ad libitum upon arrival until sacrifice. The animals were randomized into 4 groups: LFD sham, HFD sham, LFD burn, and HFD burn. A well-established method was used to induce a full-thickness scald burn of 20% TBSA^45^. Second IPGTT was performed on post-burn day 6 and all the animals were sacrificed on post-burn day 7.

### Plasma and tissue collection

Blood was collected from portal vein and cardiac puncture, respectively. Blood and liver tissue were processed as previously described^15^.

### Western blotting

Antibodies against p-ACC (Ser79), ACC, FASN, CPT1A, IP3R1, VDAC, phospho-Akt (Ser473), Rictor, MFN2, BiP, CHOP, Caspase-1, cleaved Caspase-1, cleaved Caspase-3, PARP, and GAPDH were purchased from Cell Signaling (Danvers, MA, USA). Anti- NLRP3, anti-XBP-1, and anti-ATF6 antibodies were purchased from EMD Millipore (Billerica, MA, USA). Anti-IP3R3 antibody was purchased from BD Biosciences (San Jose, CA, USA). Clarity Western ECL substrate was purchased from Bio-Rad (Hercules, CA, USA). Liver homogenates (50 μg of protein per well) were separated by 10% SDS-PAGE gel, proteins were transferred to nitrocellulose membrane as previously described^15^, and then blots were probed using the antibodies listed above. Band intensities were detected, normalized and quantified with the Chemidoc and Image Lab 5.0 software (Bio-Rad Laboratories, Hercules, CA). GAPDH was used as loading control.

### In-gel mitochondrial ETC activity assays

In-gel mitochondrial ETC activity assays were performed as described previously^23,46^.

### Immunofluorescent multi-channel staining of liver

Antibody staining was performed as described previously^15,47^. Primary antibodies were the same as in Western blotting. The percentage of marker-positive cells was determined by taking representative images and directly counting cell number by blindfolded third party. Cell enumerations for each experiment are listed in the text or figure legends.

### Hematoxylin and eosin (H&E) staining and transmission electron microscopy (TEM) of tissue sections

Liver tissue was fixed, sectioned, and stained as described previously^15,47^.

### Determination of free fatty acids (FFA), glycerol and triglyceride levels in blood

Levels of FFA, glycerol and triglyceride in the blood were determined using FFA, glycerol and triglyceride colorimetric assay kits according to the manufacturer’s instructions (Cayman Chemical, Ann Arbor, Michigan, USA).

### Statistical analysis

The statistical analysis was performed using Prism version 5.01 (GraphPad Software, San Diego, CA). One-way ANOVA with Bonferroni’s Multiple Comparison Test was used unless otherwise specified and *P* < 0.05 was considered statistically significant.
